# Tyrosine Kinase Inhibitors Induced Thyroid Dysfunction: A Review of Its Incidence, Pathophysiology, Clinical Relevance, and Treatment

**DOI:** 10.1155/2013/725410

**Published:** 2013-10-27

**Authors:** Hala Ahmadieh, Ibrahim Salti

**Affiliations:** Division of Endocrinology, Department of Internal Medicine, American University of Beirut-Medical Center, Riad El-Solh, P.O. Box 11-0236, Beirut, Lebanon

## Abstract

Tyrosine kinase inhibitors (TKI) belong to a new class of molecular multitargeted anticancer therapy which targets different growth factor receptors and hence attenuates cancer cell survival and growth. Since their introduction as adjunct treatment for renal cell carcinoma and gastrointestinal stromal tumors (GIST), a number of reports have demonstrated that TKI can induce thyroid dysfunction which was especially more common with sunitinib maleate. Many mechanisms with respect to this adverse effect of tyrosine kinase inhibitors have been proposed including their induction of thyroiditis, capillary regression in the thyroid gland, antithyroid peroxidase antibody production, and their ability to decrease iodine uptake by the thyroid gland. Of interest is the observation that TKI-induced thyroid dysfunction may actually be protective as it was shown to improve overall survival, and it was suggested that it may have a prognostic value. Followup on thyroid function tests while patients are maintained on tyrosine kinase inhibitor is strongly recommended. When thyroid dysfunction occurs, appropriate treatment should be individualized depending on patients symptoms and thyroid stimulating hormone level.

## 1. Introduction

Tyrosine kinase inhibitors (TKI), being analogues of ATP, compete with the ATP binding site of several oncogenic tyrosine kinases, hence blocking their signaling pathways involved in the phosphorylation of cellular signaling proteins which is essential for tumor cell survival and proliferation. Hence, they belong to a novel class of molecular multi targeted anticancer therapy, with significant antiproliferative and antiangiogenic activities [1, 2].

Some of the solid tumors and hematological malignancies are partially affected by abnormalities in the regulation of the tyrosine kinase related receptors such as the stem-cell factor receptor (KIT) (e.g., in gastrointestinal stromal tumor), platelet-derived growth factor receptor (e.g., in dermatofibrosarcoma protuberans), and fetal liver TK receptor 3 (FLT3) (e.g., in acute myelogenous leukemia) [3, 4].

Tyrosine kinase inhibitors, through targeting tyrosine kinase domains of platelet-derived growth factor receptor *α*/*β*, vascular endothelial growth factor receptors 1, 2, and 3, mitogen-activated protein kinase (MAPK)/extracellular signal-regulated kinases (ERK), and v-raf murine sarcoma viral oncogene homolog B1 (BRAF) inhibit the proliferation, migration, differentiation, and survival of cancer cells [5–9]. Other targets of TKI are the RET and KIT, which are also involved in the tumor growth. Although all tyrosine kinase inhibitors are known to act through the same mechanism, they do differ in their spectrum of targeted kinases, their pharmacokinetics, and their adverse effects.

Several multitarget tyrosine kinase inhibitors are now in development, but the two currently approved by the US Food and Drug Administration are the sunitinib maleate (Sutent [SU11248]; Pfizer, New York, NY) and sorafenib tosylate (Nexavar [BAY 43-9006]; Bayer, West Haven, CT). Sunitinib and sorafenib were the first two to be approved for advanced renal cell carcinoma (RCC) based on the phase III trials that have demonstrated efficacy [10, 11]. In addition, sunitinib has also been approved for gastrointestinal stromal tumor (GIST) and pancreatic neuroendocrine tumors, whereas sorafenib is currently approved for hepatocellular carcinoma [12, 13]. 

Sunitinib administration involves repeated 6-week cycles with 4 weeks of treatment period (ON period) followed by 2 weeks without treatment (OFF period).

The mechanism of action of the major available tyrosine kinase inhibitors is listed in Table 1.

With the increased use of the tyrosine kinase inhibitors, several side effects began to emerge, among which was the thyroid dysfunction, which is now recognized as being an important but potentially manageable side effect of such therapy. 

## 2. Tyrosine Kinase Inhibitors Induced Thyroid Dysfunction

### 2.1. Incidence

Table 2 lists the studies that found the association between the different tyrosine kinase inhibitors and thyroid dysfunction [14–29].

This association of sunitinib with hypothyroidism was originally noted in 2005. This led to the conduction of the first prospective observational cohort study that included patients with normal baseline TSH, observed while on sunitinib, for thyroid dysfunction, for a median period of 37 weeks. It was found that 60% had thyroid dysfunction, without any biochemical evidence of autoimmune thyroid disease. In addition, 40% had suppressed TSH prior to the hypothyroidism, suggesting that thyroiditis could have been the possible mechanism for the subsequent development of hypothyroidism. In addition, two patients in that study were found to have no evidence of thyroid tissue on thyroid ultrasound [14]. 

Since then, several other observational studies documented sunitinib-induced hypothyroidism, two of which were retrospective. (Table 2) The incidence of sunitinib-induced hypothyroidism ranged between 53% and 85% [15, 16]. 

Another prospective observational study by Mannavola et al. evaluated the effects of sunitinib in 24 patients with gastrointestinal stromal tumors. 46% of those patients developed permanent hypothyroidism, and 25% had transient hypothyroidism after the first cycle of sunitinib therapy (cycle 1 out of 6). TSH became elevated at the end of sunitinib cycle and was back to normal during the OFF treatment phase. After permanently discontinuing sunitinib, serum TSH concentrations did return to normal within 60 days in all patients. However with subsequent cycles, the degree of TSH elevation increased. No alterations in ultrasound findings and antithyroid antibodies were noted. Moreover, ^123^I uptake was significantly reduced at the end of the 4-week ON period and improved again by the end of the OFF periods [17]. 

Wolter et al. performed a prospective observational study, whose design was one of the best of all designs in the aforementioned studies, since thyroid function tests were performed before sunitinib was started and on days 1 and 28 of each treatment cycle. It was found that 61% had a transient or permanent elevation in their TSH, of whom 27% required hormone replacement. The median time for development of thyroid dysfunction was around 4 weeks. Patients who did not develop hypothyroidism within the first cycles did not do so later during therapy [18].

A recent meta-analysis of seven randomized trials which included 2787 subjects revealed a risk ratio of all- and high-grade hypothyroidism of 13.95 (95% CI 6.91–28.15; *P* < 0.00001) and 4.78 (95% CI 1.09–20.84; *P* = 0.04), respectively. Significantly higher incidence of all-grade hypothyroidism was noted in subgroup analysis of patients receiving sunitinib for longer duration as compared to those receiving it for shorter duration (*P* = 0.02) [20].

Sorafenib is another oral tyrosine kinase inhibitor, which targets multiple kinases including BRAF, VEGFR, and RET and is approved for the treatment of advanced RCC and unresectable hepatocellular carcinoma in addition to being evaluated in other tumors including lung, pancreatic, prostate, melanoma, and differentiated thyroid cancer. Incidence of thyroid dysfunction with sorafenib was much less when compared to sunitinib, ranging between 20 and 36% as evaluated in different studies [21–23]. 

Imatinib, a third commonly used oral tyrosine kinase inhibitors, currently approved for the treatment of chronic myeloid leukemia (CML), GIST tumors, and dermatofibrosarcoma protuberans and is being evaluated for other tumors including medullary thyroid cancer (MTC). In a study by de Groot et al., of 11 patients (1 GIST, 10 MTC) who received imatinib, eight had previously undergone thyroidectomies and were on thyroid hormone therapy, and those patients needed increased thyroid hormone requirements while on imatinib treatment [24, 25]. In another study by de Groot et al. out of 15 patients with advanced MTC who received imatinib, 9 of which (90%) had previously undergone total thyroidectomies and were on thyroid hormone replacement, all of which had increased thyroid hormone requirements while on therapy. On the other hand, patients with intact thyroid glands remained euthyroid while on imatinib [26]. Therefore, both studies showed that all patients with intact thyroid glands receiving imatinib had no thyroid dysfunction.

Dasatinib is a second-generation TKI used in the treatment of imatinib-resistant Philadelphia-positive CML. Kim et al. retrospectively reviewed thyroid function in ten patients who received dasatinib, 2 of which were on levothyroxine prior to starting therapy, 5 of which (50%) developed hypothyroidism (4 subclinical, 1 clinical), and two (20%) had subclinical hyperthyroidism, none of which required treatment [27].

Axitinib is another oral, potent, and selective inhibitor of vascular endothelial growth factor receptors (VEGFRs) 1, 2, and 3. The first study to report an inappropriate elevation of serum TSH levels was in 6 patients treated with axitinib for metastatic renal cell carcinoma where, within one month, five patients had elevations in TSH and 4 had suppressed TSH followed by its increase [28]. Data was reported on 18 Japanese patients who received axitinib therapy for advanced solid tumors in two phase I trials, 16 of which (89%) experienced TSH elevation [29]. Another phase I study evaluated the effects on axitinib therapy for 12 patients with advanced solid tumors, 11 of which experienced TSH elevation [30]. Increased TSH levels were highly correlated with exposure to axitinib. Axitinib effect was compared to sorafenib in a phase III trial in 723 patients with metastatic renal cell cancer, and the reported adverse events including hypothyroidism were more commonly reported with axitinib therapy, but the exact number of cases was not adequately reported (25%) [31].

Pazopanib is another multityrosine kinase inhibitor, targeting VEGFR 1, 2, and 3, c-kit, and platelet-derived growth factor receptor (PDGF-R), approved for the treatment of advanced RCC. The incidence and severity of thyroid dysfunction were explored in 578 pazopanib-treated patients who were participating in 3 trials, and it was found that 37 patients (6%) had elevated TSH at baseline (>5 MU/L), 167 (29%) had a TSH value of >5 MU/L during treatment; 97 (17%) had TSH values of >5–10 MU/L during treatment; 45 (8%) had TSH values of >10–20 MU/L; and 25 (4%) had TSH values of >20 MU/L. Hypothyroidism (TSH > 5–10 MU/L, and T4 < lower limit normal (LLN)) was also observed in 19 (3%) patients Moreover, hypothyroidism with TSH > 10 MU/L and T4 < LLN was diagnosed in 15 (3%) patients. Hyperthyroidism, defined as TSH < 0.3 MU/L and T4 > upper limit normal (ULN), was seen in 8 (1%) patients [32]. Pazopanib was evaluated in a phase III trial for locally advanced or metastatic renal cell carcinoma, and it was found that the incidence of hypothyroidism was rare, less than 10% [33]. 

Tivozanib blocks the activation of VEGFR 1, 2, and 3 more potently than earlier tyrosine kinase inhibitors. Incidence of hypothyroidism was higher in the tivozanib arm (13 subjects, 5.0%) than in the sorafenib arm (6 subjects, 2.3%) in the TIVO-1 phase III trial. TSH levels that were normal prior to dosing but increased to >10 mIU/L during treatment were reported for 30.1% of tivozanib subjects versus 7.0% of the patients who received sorafenib. A smaller number of tivozanib subjects had low free T3 or free T4 on or after the date that the elevations of TSH were observed (8.9% with low T3; 1.9% with low free T4), consistent with the occurrence of hypothyroidism AEs. It is important that the incidence of hypothyroidism for sorafenib reported in this phase III trial was lower as compared to that in earlier studies, where it was estimated to be around 20–36%. The explanation to that is not very clear [34].

Other tyrosine kinase inhibitors, including motesanib diphosphate and vandetanib which are used for variety of tumors, have also been associated with thyroid dysfunction, but it was found to occur less often [35, 36].

Little is known about the incidence of tyrosine kinase inhibitors induced thyrotoxicosis and destructive thyroiditis. Cases were reported of patients on sunitinib who developed transient overt thyrotoxicosis followed by hypothyroidism [37, 38]. Sorafenib induced thyroiditis was also reported in two patients with hepatocellular carcinoma which was later followed by overt and subclinical hypothyroidism with ultrasonography showing an atrophic thyroid gland in the first patient and signs of thyroiditis in another patient [39]. Another case was reported of hyperthyroidism and thyroid autoimmunity induced by sorafenib in patients with metastatic renal cell cancer [40] (Table 3).

### 2.2. Mechanisms Explaining Association of Thyroid Dysfunction

It is still unclear why sunitinib in particular as compared to the other tyrosine kinase inhibitors was the one to be found mostly associated with thyroid dysfunction. The possible mechanisms were recently reviewed by Makita et al. [41].

Sunitinib-induced destructive thyroiditis was one of the proposed mechanisms based on the study of Desai et al., where 40% of patients, who were hypothyroid during the first cycles of therapy, had evidence of suppressed TSH prior to the hypothyroidism [14]. Moreover, Grossmann et al. documented the presence of lymphocytic thyroiditis on fine needle aspiration of patients developing thyroid dysfunction while on sunitinib, which further supported the diagnosis of destructive thyroiditis [42].

Others hypothesized that sunitinib, through the inhibition of vascular endothelial growth factor receptor (VEGFR), led to capillary regression, since VEGF and VEGFRs are expressed by thyroid follicular cells and are, partly, regulated by TSH [43–47]. Studies in mice have shown that inhibition of VEGFR led to a 68% reversible reduction in thyroid vasculature thus damaging thyroid structure and leading to alteration of the thyroid function [48]. Moreover in the recently published review, it was hypothesized that sunitinib, through its inhibition of VEGFR signaling pathway, may reduce the thyroid micropapillary and vascular lumen leading to tissue ischemia and decreasing blood flow to the thyroid. This might explain the reason why sunitinib in particular was the one mostly associated with thyroid dysfunction. It was suspected that not only VEGFR 2 but also VEGFR 1 and PDGFR may need to be inhibited to cause thyroid ischemia. Hence, more potent and specific TKIs that target the VEGFRs and PDGFR such as sunitinib would lead more frequently to thyroid dysfunction [20]. Aside from TKIs, it is well known that the humanized monoclonal antibody bevacizumab also targets VEGF signaling and therefore prevents binding of VEGF to its receptors, and it has also been approved for the treatment of renal cell, colorectal, breast, and non-small-cell lung cancer (NSCLC) [49]. Bevacizumab has fewer targets than tyrosine kinase inhibitors, and it was found that the incidence of bevacizumab-induced hypothyroidism is low when compared to sunitinib, but still the effects of bevacizumab on the thyroid gland cannot be underestimated. It was found to decrease vascular permeability and led to capillary vasoconstriction in the thyroid [50, 51]. However, the lower incidence of thyroid dysfunction was mainly attributed to the potential protective effect of placenta growth factor (PlGF), which was found not to be targeted by bevacizumab, thus restoring the vascularity in the thyroid gland [52]. Apparently patients with preexistent thyroid disorders are more susceptible to bevacizumab treatment [53].

Another hypothesis was proposed by Mannavola et al., where a significant reduction of iodine uptake was found during the ON period of sunitinib therapy, which was reversible during the OFF periods [17]. Therefore, iodine uptake blocking could be involved as a possible mechanism. However, until now, no effect of sunitinib on iodine uptake or on sodium iodide symporter has been demonstrated. In addition, one *in vitro* study demonstrated the contrary [47].

The fourth mechanism proposed by Wong et al. was the antithyroid peroxidase antibody (anti-TPO) activity induced by sunitinib, which could explain the latent period between sunitinib use and development of hypothyroidism [16]. However in the study by Mannavola et al., anti-TPO antibodies remained negative in all cycles in patients who had normal thyroid function tests at baseline [17]. The study by Wolter et al. found positive antithyroid antibodies in only a minority of patients who had high TSH [18].

The effect of sorafenib on thyroid physiology is not yet understood. Sorafenib was also shown to inhibit VEGFR and PDGFR signaling pathways. It may also interact with TSH-signaling pathways which are thought to involve RAF pathway [7, 48]. The studies on imatinib and thyroid dysfunction cannot clearly identify the way imatinib acts on the thyroid gland [25, 26].

### 2.3. Clinical Implication of Tyrosine Kinase Inhibitor Induced Thyroid Dysfunction

Although TKI-induced hypothyroidism is an unwanted side effect of TKI therapy, data suggested that it may have an important prognostic value. Wolter et al. noted that the median progression-free survival and overall survival in patients who developed hypothyroidism on sunitinib therapy were much better (10.3 months) as compared to patients who remained euthyroid (3.6 months) [54]. Another small Japanese study noted that tumor response rate was 73% in patients who developed hypothyroidism while being treated with sunitinib for renal cell carcinoma, as compared to 33% tumor response rate in patients who remained euthyroid [55]. Clinical efficacy was also evaluated by Schmidinger et al. in their prospective analysis of 87 patients with metastatic RCC who are being treated with sunitinib or sorafenib. Subclinical hypothyroidism was present in five patients at baseline and developed in 30 patients (36.1%) within the first 2 months of therapy. It was noted that patients who developed subclinical hypothyroidism had a statistically significant better objective remission rate of 28.3% as compared to 3.3% remission rate in euthyroid patients (*P* < 0.001) [23]. This was again demonstrated in another multivariate analysis by Riesenbeck et al., where hypothyroidism was associated with a longer progression free survival (16.0 ± 0.8 months versus 6.0 ± 0.8 months, *P* = 0.032) and therefore was found to be an independent prognostic predictor of survival (*P* = 0.01) [24]. As per the recent review by Makita and Iiri, it was noted that patients developing thyroid dysfunction might tend to have their cancer better controlled through the common antiangiogenic effects affecting both the tumor and the thyroid [41].

It is important to note that there has been an observed correlation between the development of hypothyroidism and tyrosine kinase inhibitors use, but several questions are still unanswered, such as whether the time course to hypothyroidism would predict response to cancer therapy, and whether developing hypothyroidism soon after therapy or later on would affect clinical response. Moreover, it is still not known whether the degree of TSH elevation would impact efficacy.

## 3. Treatment of Tyrosine Kinase Inhibitors Associated Thyroid Dysfunction

Although it has already been known that TKIs are associated with thyroid dysfunction and even had a prognostic value, there are still no guidelines with regard to the frequency of thyroid function tests monitoring and treatment of thyroid dysfunction. Serum TSH concentration on day 1 of each cycle has been suggested as a reasonable guideline, but this has not been proven [56].

Wolter et al. proposed measuring TSH on days 1 and 28 of the first 4 cycles of sunitinib because most thyroid function tests abnormalities were noted to occur in the first few cycles. He also proposed that patients with normal TSH values after the first 4 cycles can have their TSH measured on day 28 of every 3rd cycle [18]. Patients with preexisting thyroid abnormalities, on thyroid hormone replacement, may need increased requirements [57, 58]. As for the level of TSH elevation that warrants treatment, expert groups such as the American Thyroid Association, the American Association of Clinical Endocrinologists, and the Endocrine Society recommend thyroid hormone to be initiated whenever TSH rises to a level above 10 uIU/mL, but data is lacking with regard to treatment of TSH between 4.5 and 10. Wolter et al. recommended thyroid hormone replacement if TSH is elevated to a level above 10 uIU/mL on day 1 of a cycle of sunitinib therapy, and he warned against thyroid hormone replacement based on results of thyroid function tests on day 28, since this may lead to overtreatment at the end of treatment [18]. In addition, Garfield et al. cautioned against the routine use of thyroid hormone replacement, since it may lead to tumor growth in patients with active cancers especially with the data that showed that hypothyroidism in such patients was protective [59].

However a study by Bladou et al. compared progression free survival in patients treated for thyroid dysfunction developing with sunitinib therapy as compared to those not treated and noted that progression free survival was not different between the two groups (*P* = 0.94). Therefore, the relationship between exogenous thyroid hormone and cancer growth is still not very well established. The association requires more trials to further investigate whether thyroid hormone replacement would affect cancer outcomes [60].

## 4. Conclusion

Tyrosine kinase inhibitors are the new molecular targeted therapies approved for the treatment of several hematological and solid tumors. Many studies clearly have demonstrated that they were able to induce thyroid abnormalities including hypothyroidism and less often hyperthyroidism. Increased awareness and monitoring of thyroid function tests are important in patients maintained on TKIs. Several mechanisms have been proposed for this tyrosine kinase inhibitors associated thyroid dysfunction. There are still no clear established guidelines with regard to the treatment of thyroid dysfunction induced by tyrosine kinase inhibitors.

## Figures and Tables

**Table 1:** Mechanisms of action of different tyrosine kinase inhibitors.

Major tyrosine kinase inhibitors	Mechanism of action
Sunitinib	Inhibition of VEGF 1–3, PDGF Ra/*β*, KIT, FLT3-ITD, FLT3, and Ret
Sorafenib	Inhibition of vascular endothelial growth factor receptor 2 (VEGFR 2), platelet-derived growth factor receptor (PDGFR), FLT3, Ret, and c-Kit
Imatinib	Inhibition of Bcr-Abl positive colonies from CML patients, platelet-derived growth factor (PDGF), stem cell factor (SCF), and c-Kit
Dasatinib	Inhibition of Bcr-Abl and Src family kinases (SFK)
Axitinib	Inhibition of VEGFR 1, 2, and 3 selectively
Motesanib	Inhibition of VEGFR, PDFGRs, KIT, and RET
Nilotinib	Inhibition of BCR-ABL
Pazopanib	Inhibition of VEGFR 1, 2, and 3, c-kit, and platelet-derived growth factor receptor (PDGFR)

**Table 2:** Incidence and prevalence of tyrosine kinase inhibitor associated thyroid dysfunction.

Authors (year)	Study type	TKI drug type	Number of patients	Tumor type	Patients with hypothyroidism (↑TSH) (%)	Patients with isolated suppressed TSH (%)	Patients with suppressed TSH prior to elevated TSH (%)
Rini et al. [15] (2007)	Retrospective	Sunitinib	66	RCC	56 (85%)	None	None

Wong et al. [16] (2007)	Retrospective	Sunitinib	40	Solid (mostly GIST)	21 (53%)	None	None

Desai et al. [14] (2006)	Prospective	Sunitinib	42	GIST	Persistent = 15 (36%) Transient = 7 (17%)	4 (10%)	6 (14%)

Mannavola et al. [17] (2007)	Prospective	Sunitinib	24	GIST	Total = 17 (71%) Persistent = 11 (46%)	None	None

Wolter et al. [18] (2008)	Prospective	Sunitinib	59	RCC, GIST	Total = 36 (61%) 16 (27%) required thyroid hormone treatment 20 (34%) did not require thyroid hormone treatment	None	None

Shinohara et al. [19] (2011)	Prospective	Sunitinib	17	RCC	8 (47%)	None	None

Tamaskar et al. [21] (2008)	Retrospective	Sorafenib	39	RCC	7 (18%)	1 (2.6%)	None

Clement et al. [22] (2008)	Prospective	Sorafenib	38	RCC	7 (30%)	1 (5%)	None

Schmidinger et al. [23] (2011)	Prospective	Sunitinib or sorafenib	87	RCC	5 (5.7%) subclinical hypothyroidism in 30 patients (36.1%) after 2 months of treatment	None	None

Riesenbeck et al. [24] (2011)		Sunitinib or sorafenib	66	RCC	21 (31.8%)	None	None

de Groot et al. [25] (2007)	Prospective	Imatinib	11	MTC, GIST	8 out of 8 patients with previous thyroidectomies had increased thyroid hormone requirement Other patients were euthyroid	None	None

de Groot et al. [26] (2005)	Prospective	Imatinib	15	MTC	9 out of 10 patients with previous thyroidectomies had increased thyroid hormone requirement Other patients were euthyroid	None	None

Kim et al. [27] (2010)	Prospective	Nilotinib	55	Ph-positive CML	Total = 12 (22%) Transient = 8 (15%) Persistent = 4 (7%)	Total = 18 (33%) Transient = 15 (27%) Persistent = 3 (5%)	None

Sherman et al. [35] (2008)	Prospective	Motesanib diphosphate	99	DTC	17 (22%) (all patients athyreotic)	None	None

Robinson et al. [36] (2010)	Prospective	Vandetanib	19	MTC	Mean 5.1-fold increase in TSH in 17 patients with available TFTs	None	None

Robinson et al. [36] (2010)	Prospective	Axitinib	18	Solid tumors	16 (89%)	None	None

Ohba et al. [28] (2013)	Prospective	Axitinib	6	Metastatic renal cell carcinoma	5 (83%)	1 (16%)	4 (66.6%)

Fujiwara et al. [29] (2012)	Prospective	Axitinib	18	Advanced solid tumors	16 (89%)	None	None

Mukohara et al. [30] (2010)	Prospective	Axitinib	12	Advanced solid tumors	11 (92%)	None	None

Wolter et al. [32] (2011)	Prospective	Pazopanib	578	Renal cell carcinoma	Elevated TSH at baseline (>5 MU/L) was 37 (6%). 167 (29%) patients had a TSH value of >5 MU/L during treatment; 97 (17%) patients had TSH values of >5–10 MU/L during treatment; 45 (8%) patients had TSH values of >10–20 MU/L; and 25 (4%) patients had TSH values of >20 MU/L. Hypothyroidism (TSH > 5–10 MU/L, and T4 < LLN) was observed in 19 (3%) patients. Hypothyroidism with TSH > 10 MU/L and T4 < LLN was diagnosed in 15 (3%) patients	Hyperthyroidism, defined as TSH < 0.3 MU/L and T4 > ULN, was seen in 8 (1%) patients	None

Sherman et al. [35] (2008)	Prospective (phase III trial)	Pazopanib	202	Locally advanced or metastatic renal cell carcinoma	<10%	None	None

Motzer [34] (2013)	Prospective (phase III trial)	Tivozanib	260	Advanced renal cell carcinoma	Preliminary results: the incidence of hypothyroidism was higher in the tivozanib arm (13 subjects, 5.0%) than in the sorafenib arm (6 subjects, 2.3%)	None	None

**Table 3:** Frequency of reported hypothyroidism and hyperthyroidism for different tyrosine kinase inhibitors used.

Tyrosine kinase inhibitors	Frequency of hypothyroidism	Frequency of isolated hyperthyroidism
Sunitinib	53–85%	10%
Sorafenib	20–36%	2.6–5%
Imatinib	90–100% (only in patients who underwent previous thyroidectomies)	0%
Motesanib diphosphate	22%	0%
Vandetanib	89%	0%
Axitinib	83–92% (small number of patients in studies included)	16%
Pazopanib	10–29%	0%
Tivozanib	5%	0%
